# Mental fatigue impairs padel‐specific psychomotor performance in youth‐elite male players

**DOI:** 10.1002/ejsc.12039

**Published:** 2024-03-18

**Authors:** Jesús Díaz‐García, Jelle Habay, Ana Rubio‐Morales, Jonas de Wachter, Tomás García‐Calvo, Bart Roelands, Jeroen Van Cutsem

**Affiliations:** ^1^ Faculty of Sport Sciences University of Extremadura Cáceres Spain; ^2^ Human Physiology and Sports Physiotherapy Research Group Vrije Universiteit Brussel Brussels Belgium; ^3^ VIPER Research Unit Royal Military Academy Brussels Belgium

**Keywords:** exercise, motivation, overtraining, performance, psychology

## Abstract

While the interest and professionalization of padel around the world, and the importance of mental fatigue in sport science literature are both on the rise, not much is known about how mental fatigue impacts padel‐specific performance. Forty‐eight male elite youth players (18 ± 2 y) participated in this randomized counterbalanced crossover study. The players visited the laboratory on three separate occasions: familiarization, control (i.e., 30‐min documentary), and mental fatigue (i.e., 30‐min incongruent Stroop) condition. The participants' perceived mental fatigue, vigilance (i.e., Psychomotor Vigilance Test) and response inhibition (i.e., Stroop task) were assessed as markers of mental fatigue. The Padel accuracy was evaluated using a validated padel‐specific performance task that includes the usage of different strokes (i.e., drive, drive volley, bandeja, and drive attack after the use of the glass). All these variables were assessed pre‐ and post‐ both the control and mentally fatiguing task. A condition × time interaction was found for perceived mental fatigue, the Stroop performance and PVT reaction time (*p* < 0.001) and for padel‐specific accuracy on all strokes (*p* < 0.001). The perceived level of mental fatigue increased, while PVT reaction time and Stroop performance (i.e., amount of words) were impaired in time only in the mental fatigue condition. The accuracy of all padel strokes was significantly impaired from pre to post only in the mental fatigue condition (*p* < 0.050). In conclusion, mental fatigue impairs padel‐specific accuracy in young elite male padel players. Consequently, we recommend coaches to consider the level of mental fatigue during padel trainings and competitions.

## Highlights

- The result of the present study shows that mental fatigue induced by a 30‐min incongruent Stroop task, impaired the accuracy and the speed of different padel‐specific strokes in youth‐elite male players.
- The presence of mental fatigue during official padel competitions has been previously confirmed. Then, it might of course impact the outcome of the match, as the number of forced and unforced errors or the speed of the shots are important indicators of padel‐specific performance.
- This information highlights the need for coaches and staff to consider the amount of mental fatigue generated during padel trainings and matches. Coaches are able to modulate the level of mental fatigue of the trainings.

## 1 INTRODUCTION

Sports situations pose very high demands on all aspects of the human body, such as the cardiorespiratory or neuromuscular system, and certainly also the brain. Therefore, athletes may feel physically as well as mentally fatigued while performing their respective sport (Van Cutsem & Marcora, 2021). Physical fatigue has been a widely investigated topic in sport science for many years. In contrast, the interest in mental fatigue has only emerged recently (Graham & Brown, 2021). Mental fatigue can be defined as a psychobiological state induced by demanding cognitive or emotional tasks, and can be experienced subjectively, behaviorally and/or physiologically, depending on the individual (Boksem & Tops, 2008; Russell et al., 2020). Mental fatigue has been shown to impair both endurance as well as sport‐specific psychomotor performance, while anaerobic performance is mostly non‐influenced (Habay, Van Cutsem, et al., 2021; Van Cutsem et al., 2017). Hence, mental fatigue becomes an important issue in the sport performance context.

The sport‐specific cognitive efforts (e.g., sustained attention to analyze the game‐scenario) and the control of emotions (e.g., the control of negative emotions such as anxiety) differ between different sports or situations within the same sport (Russell et al., 2020). It could be argued that, the more emotionally and cognitively demanding a particular sport is, the more it might trigger mental fatigue in game situations. One type of sports that features a high contribution of cognitive resources are racket sports, where similar to other sports they require specific tactical decisions, temporal pressure or high levels of stress, factors that may all contribute to mental fatigue (Le Mansec et al., 2018). In line with this, the current study is focused on padel, a racket sport practiced worldwide which has seen an intensive growth in recent years (Courel‐Ibañez et al., 2019). Although there is not a lot of data available to support this, the ranking of the International Padel Federation shows that in the last 10 years the number of professional padel players has increased 10‐fold. It is estimated that around eight million people around the world practice padel nowadays. For example, in Spain, the Government Sport Organization reported that padel is the second most practiced sport in the country, and the number of padel practitioners has evolved from ten thousand practitioners approximately in 2010 to 2 million in 2019. In fact, padel has become globalized and as an example, the professional circuit that previously took place in Spain, now includes professional events in Africa (e.g., Egypt), Asia (e.g., Qatar), South America (e.g., Argentina, Brazil and Chile), North America (e.g., United States of America), and Europe (e.g., Italy, Belgium and Sweden) (Díaz‐García, González‐Ponce, et al., 2021).

Padel is a racket sport characterized by a specific court (which includes a glass and metal wall where the ball can bounce off, increasing the entropy of the game (Courel‐Ibáñez et al., 2019)), and the presence of a teammate (which results in the need to maintain emotional interdependence (Díaz‐García, González‐Ponce, et al., 2021)). It is therefore no surprise that cognitive and emotional demands, triggered during padel matches, cause a significant increase in the self‐reported level of mental fatigue in professional padel players (Díaz‐García, González‐Ponce, et al., 2021).

Despite this information, to the best of our knowledge, no study has yet investigated the effects of mental fatigue on padel‐specific performance. This can create a ton of practical applications and research opportunities, such as allowing padel coaches to study the effectiveness of recovery strategies for mental fatigue (Díaz‐García, González‐Ponce, et al., 2021), or deciding if it would be best to avoid the presence of mentally fatiguing scenarios close to competitions or games. Therefore, the present study aimed to investigate the effects of mental fatigue on padel‐specific performance. We hypothesized that mental fatigue would impair padel‐specific performance, based on previous research investigating effects of mental fatigue in different racket sports (Le Mansec et al., 2018; Van Cutsem et al., 2019).

## 2 METHOD

### 2.1 Participants

An a priori sample size calculation based on the results reported in a previous study that focused on the effects of mental fatigue on the performance of another racket sport (i.e., table tennis) (Le Mansec et al., 2018) (*η*_*p*_^2^ = 0.244) showed that a total of 22 participants were needed to observe the effect of mental fatigue on sport‐specific racket performance. This sample size calculation was performed following the recommendations of Lakens (2022). A total of 48 male elite youth players (*M*_age_ = 18, *SD*_age_ = 2, *M*_WPTranking_ = 186, *SD*_WPTranking_ = 15) voluntarily participated in the study. All players had frequently participated in the International Youth Championships and World Padel Tour before inclusion in the present study. In addition, all the participants completed padel training sessions for at least 3–4 days a week with 3.5 h of training per day (1 h of physical training, 1 h of technical training and 1.5 h of padel matches).

All participants provided written informed consent before the start of the study. The experimental protocol and procedures were approved by the Extremadura's University Ethics Local Committee (approval number: 93/2020). The participants were blinded to the aim of the study and were told that the current study aimed to evaluate the impact of different cognitive activities on padel‐specific performance, without emphasizing that we expected negative effects of performing the Stroop task on padel‐specific performance.

### 2.2 Experimental design

**FIGURE 1:**
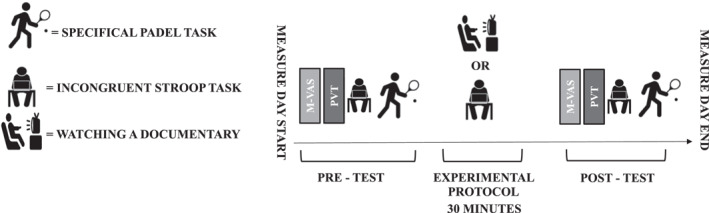
Timeline of testing procedures. PVT, Psychomotor Vigilance Task; VAS, Visual Analogue Scale.

They visited the laboratory on three separate occasions (Familiarization, Control condition (documentary) and Intervention condition (Stroop task)). All the visits took place at the same time of day. The total duration of each visit was approximately 1 h. Participants were encouraged to sleep at least 7 h in the previous nights, refrain from caffeine and alcohol in the 12 h before each visit to the laboratory, refrain from creatine during the study, and not to practice vigorous physical activity the 24 h before each visit to the laboratory. Figure 1 indicates the timelines to assess the effects of induced mental fatigue on perceptions of mental fatigue, Stroop performance, and padel‐specific performance.

Mental fatigue was induced using a 30‐min incongruent Stroop (UMH‐MEMTRAIN). The interstimulus interval was 1900 ms, and the maximum duration allowed for answering was not defined although participants were encouraged to answer as fast and accurately as possible. During the Stroop task, a member of the research team sat behind the participants to ensure participants stayed focused on the task. The Stroop was performed on a 69 × 55 cm monitor, and participants were seated 65 cm away from the monitor. Participants were instructed to provide a response by pressing specific buttons. The Stroop task is a self‐regulation task where different words representing colors (blue, green, red, and yellow) are printed in different colors on a black background. Subjects were instructed to indicate the meaning of the words as quickly and accurately as possible. All the words were presented in a different color than their meaning (100% incongruent). The Stroop task has been one of the most used tasks to induce mental fatigue in studies analyzing the effects of mental fatigue in sports (Van Cutsem et al., 2017). In fact, the decision to use 30‐min of Incongruent Stroop to induce mental fatigue was based on previous studies showing that this duration is sufficient to achieve this purpose (Ishii et al., 2014; Pageaux & Lepers, 2018).

### 2.3 Instruments and outcome measures

#### 2.3.1 Mental fatigue visual analog scale

Subjects were asked to indicate the perceived level of mental fatigue by placing a mark on a 10‐cm line (“*How mentally fatigued do you feel*?”). The left side of the scale indicated “not at all”, while the right side indicated “maximum level of mental fatigue possible”. The research has indicated that this type of measurement is the most sensitive in detecting levels of mental fatigue (Smith et al., 2019). To improve metacognition, mental fatigue was previously defined to players as a psychobiological state caused by prolonged periods of demanding cognitive activity (Van Cutsem et al., 2017). To clarify this definition, the researchers showed participants common descriptors reported by mentally fatigued athletes (Russell et al., 2019; e.g., increased feelings of physical exertion than normal, demotivation, lack of energy, or deconcentration).

#### 2.3.2 100% incongruent stroop task

Response inhibition was assessed by a 45‐s incongruent Stroop performed on a computer. Specifically, we recorded the number of words read during these 45 s. Researchers indicated to the participants when an incorrect answer occurred, if a mistake was made the participant was instructed to repeat this word until it was correct. As such, committing errors resulted in a lower amount of total responses and a drop in performance.

#### 2.3.3 Psychomotor vigilance task

A 3‐min version of the Psychomotor Vigilance Task (PVT) was used to measure simple reaction time (i.e., vigilance) (Dinges & Powell, 1985). The interstimulus duration was random from 1 to 4 s. Subjects were required to push the screen of a mobile phone as fast as possible after a visual stimulus had appeared in the center of that screen. If the screen was pushed before the stimulus appeared, a “false start” message was presented. Mean reaction time and accuracy (i.e., amount of mistakes) during the 3‐min task were recorded.

#### 2.3.4 Padel‐specific strokes performance tests for accuracy

A previously validated instrument designed to analyze the accuracy of padel strokes was used to assess padel‐specific performance (Sánchez‐Alcaraz et al., 2016) Figure 2. A total of 4 different padel strokes were performed: (1) a drive attack stroke after the use of the glass, a stroke performed from the last part of the court and where the ball bounces off the glass and comes over the head of the player that uses this height to perform a fast stroke with the aim to win the point; (2) a drive volley, that is similar to a tennis volley, performed close to the net and without a previous bounce of the ball on the courtside of the player that performs the stroke; (3) bandeja, a specific padel stroke performed from the middle part of the court that is normally performed by the player that is close to the net and after a previous lob from the other side of the court (i.e., the players that were defending). Bandeja is performed at a height close to the head and the main purpose of this stroke is to send the ball close to the end of the court at a slow speed, so that the player that executed the shot and his/her partner can retake the net position neutralizing the opponent's lob; and (4) a drive without the use of the glass, that is similar to a tennis drive from the last part of the court and after a previous bounce of the ball on the courtside of the player that performs the stroke. The mean accuracy of 4 performed sets was obtained per specific type of stroke (i.e., participants performed 4 drive attacks, 4 drive volleys, 4 bandejas and 4 drives without glass in this order; No recovery was included within a set of 4 shots of the same type of stroke. In contrast, between sets of specific stroke types, a 20‐s recovery was included). The duration of the task was approximately 5 min. The ball was sent by a specific robot to ensure maximal standardization. The accuracy of the players was assessed by measuring the distance between the corner (i.e., the more difficult zone for defending players) and the location of the first bounce of the ball, which was then converted into a point system. Specifically, the player obtained 9 points if the first bounce was in a previously delimited zone of 3 × 3 m. From the corner; 6 points if the first bounce was in a zone of 1 × 1 just outside the 3 × 3 zone; 3 points if the first bounce was in a zone of 1 × 1 just outside the defined 6‐point zone; and 0 points if the first bounce was out of the scoring zone. Accuracy was live evaluated by an evaluator who was placed close to the scoring area. The speed of the ball was measured using a radar device that was placed in a fixed zone of the glass in front of the zone where the participants practiced (Stalker Professional Sports Rada). The calibration of the radar was performed by the researchers before the participants arrived.

**FIGURE 2:**
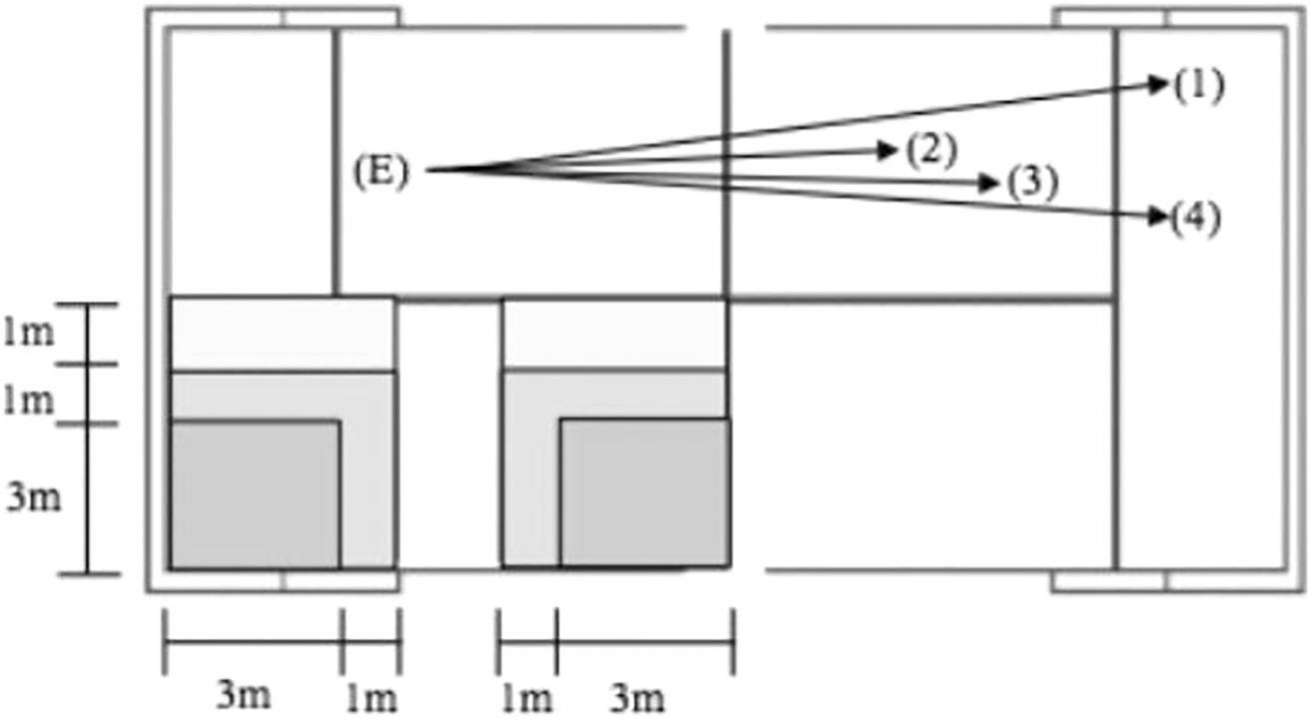
Overview of the padel specific task. Reprinted from “Groundstroke accuracy assessment in padel players according to their level of play”, by Sánchez‐Alcaraz, B.J., Courel‐Ibañez, J., & Cañas, J., 2016, *International Journal of Sport Sciences*, 12(45), 324–333. E = Coach. Strokes: (1) drive attack stroke after the use of the glass, (2) drive volley, (3) bandeja and (4) drive without the use of glass.

The participants were instructed to perform each stroke as accurately as possible. As such, in the present study, accuracy is the main outcome measure of padel‐specific performance. In terms of ball speed, no speed instruction was given to the participants. Nevertheless, ball speed was still measured via the use of a radar. Participants were blinded for the use of this radar, and were told that the speed radar was a small video camera to evaluate where the ball exactly bounced in the scoring zone. Although the combination of speed and accuracy is the most ecological way to analyze padel‐specific performance (e.g., nowadays padel players need to play as accurate as possible but also using fast strokes to win points), researchers wanted to impact the speed‐accuracy tradeoff in such a way that participants prioritized accuracy over speed. In case this prioritization choice in the speed‐accuracy tradeoff would have been left to each individual participant, this would have resulted in substantial interindividual variability and a decreased ability to evaluate the possible impact of mental fatigue on padel‐specific performance accuracy. In addition, the inclusion of a speed measure, wherefore the participants were blinded, allowed the researchers to check if the participants adopted a different stroke strategy (e.g., different motor control) to maximize their padel‐specific accuracy in both conditions.

### 2.4 Data analysis

All data are presented as means ± standard deviation (SD). The Shapiro‐Wilk test was used to test the normality of the data; sphericity was verified by Mauchly's test. When the assumption of sphericity was not met, the significance of F ratios was adjusted with the Greenhouse‐Geisser procedure. A 2 × 2 repeated measures (2 conditions—control and mental fatigue— × 2 time intervals—pre and post experimental protocols) was used to evaluate the effect of condition and time. Significant interaction effects between time and condition were followed up with pairwise comparisons. Effect sizes are reported as partial eta squared *η*_*p*_^2^ and, based on the publication of Cohen (1992), interpreted as small (0.02), moderate (0.13) and large (0.26) effects sizes. Cohen's d effect sizes were calculated for pairwise comparisons. Significance was set at < 0.05 for all analyses, which were conducted using the Statistical Package for the Social Sciences, version 25.0 (SPSS Inc.).

## 3 RESULTS

There were no significant differences between conditions in the perceived level of mental fatigue, PVT reaction time, Stroop performance and padel‐specific accuracy and speed performance, before the start of the experimental/control procedures.

### 3.1 Markers of mental fatigue

Information about markers of mental fatigue is presented in Table 1. There was a significant condition × time interaction for the perceived level of mental fatigue (*F* = 547.71; *p* < 0.001; *η*_*p*_^2^ = 0.92), PVT reaction time (*F* = 175.87; *p* < 0.001; *η*_*p*_^2^ = 0.84) and Stroop task performance (*F* = 161.76; *p* < 0.001; *η*_*p*_^2^ = 0.79). The perceived level of mental fatigue increased (*p* < 0.001; Cohen's *d* = 0.55), while PVT reaction time (*p* < 0.001; Cohen's *d* = 0.37) and Stroop performance (i.e., amount of words; *p* = 0.041; Cohen's *d* = 0.15) were impaired following the mentally‐fatiguing protocol. No significant changes were observed in perceived level of mental fatigue (*p* = 0.44; Cohen's *d* = 0.02), PVT (*p* = 0.25; Cohen's *d* = 0.01) and Stroop performance (*p* = 0.79; Cohen's *d* = 0.01) following the control condition.

**TABLE 1:** Pre‐ and post‐ treatment assessment of perceived level of mental fatigue, reaction time and heart rate.

Protocol used	VAS (a.u.)		PVT (s)		Number of words incongruent stroop (a.u)	
	Pre	Post	Pre	Post	Pre	Post
Control	1.56 ± 0.68	1.96 ± 0.22^c^	0.362 ± 0.01	0.366 ± 0.02^c^	94.23 ± 5.23	93.78 ± 4.79
Mental fatigue	1.50 ± 0.65	8.7 ± 0.83***	0.364 ± 0.01	0.440 ± 0.03***	94.88 ± 5.41	86.65 ± 5.43*^b^

*Note*: Difference between Pre and Post = *p < 0.05, **p < 0.01, ***p < 0.001; difference between post Control and post Mental fatigue = ^a^ *p* < 0.05, ^b^ *p* < 0.01, ^c^ *p* < 0.001.

Abbreviation: a.u., arbitrary units.

### 3.2 Padel‐specific accuracy and speed

Information about the effects of both conditions on padel‐specific psychomotor performance is presented in Table 2. There was a significant condition × time interaction for accuracy (*F* = 7.25, *p* < 0.001, *η*_*p*_^2^ = 0.28 for drive volley; *F* = 5.32, *p* < 0.001, *η*_*p*_^2^ = 0.26 for drive attack; *F* = 4.38, *p* < 0.001, *η*_*p*_^2^ = 0.23 for drive; *F* = 5.26; *p* < 0.001; *η*_*p*_^2^ = 0.26 for bandeja) and speed for all executed shots (*F* = 11.82, *p* < 0.001, *η*_*p*_^2^ = 0.31 for drive volley; *F* = 9.44, *p* < 0.001, *η*_*p*_^2^ = 0.30 for drive attack; *F* = 9.22, *p* < 0.001, *η*_*p*_^2^ = 0.29 for drive; *F* = 6.99, *p* < 0.001, *η*_*p*_^2^ = 0.27 for bandeja). As shown in Table 2, no change in any accuracy (*p* = 0.19, Cohen's *d* = 0.02 for drive volley; *p* = 0.21, Cohen's *d* = 0.00 for drive attack; *p* = 0.29 Cohen's *d* = 0.00 for drive; *p* = 0.22, Cohen's *d* = 0.02 for bandeja) or speed parameters (*p* = 0.18, Cohen's *d* = 0.02 for drive volley; *p* = 0.25, Cohenñs *d* = 0.01 for drive attack; *p* = 0.30, Cohen's *d* = 0.05 for drive; *p* = 0.27, Cohen's *d* = 0.04 for bandeja) was observed following the control condition. On the contrary, drive volley speed (*p* < 0.001, Cohen's *d* = 0.22) and drive volley accuracy (*p* < 0.001, Cohen's *d* = 0.17), drive attack stroke after the use of the glass speed (*p* < 0.001, Cohen's *d* = 0.36) and drive attack stroke after the use of the glass accuracy (*p* < 0.001, Cohen's *d* = 0.27), drive speed (*p* < 0.001, Cohen's *d* = 0.24) and drive accuracy (*p* < 0.001, Cohen's *d* = 0.18), bandeja speed (*p* < 0.001, Cohen's *d* = 0.25) and bandeja accuracy (*p* < 0.001; Cohen's *d* = 0.29) were all significantly impaired following the experimental condition (see Table 2).

**TABLE 2:** Pre‐ and post‐ treatment assessment of speed and accuracy of different padel‐specific strokes.

Protocol used	Volley				After‐glass				Drive				Bandeja			
	Speed (km/h)		Accuracy (a.u.)		Speed (km/h)		Accuracy (a.u.)		Speed (km/h)		Accuracy (a.u.)		Speed (km/h)		Accuracy (a.u.)	
	Pre	Post	Pre	Post	Pre	Post	Pre	Post	Pre	Post	Pre	Post	Pre	Post	Pre	Post
Control	21.05	20.66^c^	8.88	8.38^c^	40.46	40.15^c^	7.00	7.06^c^	22.66	22.33^c^	8.56	8.19^c^	19.68	19.35^c^	6.94	6.66^c^
	±1.11	±1.18	±0.61	±1.23	±1.37	±1.45	±1.43	±1.69	±2.26	±2.25	±1.07	±1.35	±1.35	±1.34	±1.41	±1.16
Mental fatigue	21.24	18.71***	8.94	7.38***	40.44	37.20***	6.69	4.25***	22.86	20.68***	8.75	7.31***	19.87	17.58***	6.31	4.50***
	±1.08	±1.13	±0.43	±1.63	±37.19	±1.48	±1.55	±1.94	±2.13	±1.89	±0.88	±1.74	±1.38	±1.12	±1.12	±2.39

*Note*: Difference between Pre and Post = *p < 0.05, **p < 0.01, ***p < 0.001; difference between post Control and post Mental fatigue = ^a^ *p* < 0.05, ^b^ *p* < 0.01, ^c^ *p* < 0.001.

Abbreviation: a.u., arbitrary units.

## 4 DISCUSSION

The purpose of the present study was to test the effects of mental fatigue on padel‐specific performance, and more specifically on the accuracy of padel‐specific strokes in youth‐elite male players. The main finding of the present study was that the mental fatigue induced by a 30‐min incongruent Stroop task, impaired the accuracy and the speed of different padel‐specific strokes in youth‐elite male players, confirming other studies investigating the effects of mental fatigue on racket sports (Habay, Proost, et al., 2021; Le Mansec et al., 2018).

### 4.1 Markers of mental fatigue

The increase in the self‐reported mental fatigue confirms that a higher state of mental fatigue was induced following the Stroop task compared to the control task in youth‐elite male players. In addition, mental fatigue‐associated impairments in PVT‐reaction time and Stroop task performance further confirm the presence of a higher mental fatigue state in the experimental condition compared to in the control condition (Van Cutsem et al., 2019). A lot of factors are known to play a role in how mentally fatiguing a certain task is perceived to be. For example, the duration of the task and the level of engagement and motivation have been shown to impact the mentally fatiguing properties of a certain task (Van Cutsem & Marcora, 2021). In the present study, considering both the perceived level as well as the behavioral markers of mental fatigue, we can conclude that we were successful in inducing mental fatigue in our participants. Although the Stroop task is not an ecologically valid padel task, this task does rely on the ability to inhibit responses, working memory and cognitive flexibility (Van Cutsem & Marcora, 2021) cognitive functions that are also relied upon during padel game scenarios (Díaz‐García, González‐Ponce, et al., 2021). The follow‐up research should also include neurophysiological measures (e.g., electroencephalography) to further, and even more firmly, confirm the presence of mental fatigue (Habay, Proost, et al., 2021).

Important to note is that the level of mental fatigue induced in the present study is higher compared to the level of mental fatigue that is perceived following the completion of a padel match (Díaz‐García, González‐Ponce, et al., 2021). This discrepancy between the level of mental fatigue that is induced by a cognitive task and by a real‐life sport game has also been observed in other sports (e.g., in soccer). Abbott et al. (2020) and Thompson et al. (2020) reported the level of perceived mental fatigue that is experienced upon completion of specific soccer match scenarios using a VAS from 0 to 100. The soccer players are indicated to perceive a level of mental fatigue of approximately 30 to 50 during match day, and around 35 to 50 during match day+1. On the other hand, Filipas et al. (2021) analyzed the effects of mental fatigue on soccer‐specific performance and reported an induced level of mental fatigue of 60 to 80 points on this same scale. In addition, in the study of Smith, Zeuwts, et al. (2016) (i.e., evaluating the effects of mental fatigue on decision making) participants exceeded 60 on a similar VAS. In the present study, the lab‐based induced mental fatigue exceeds by 3 (or 30, on the VAS scale from 0 to 100) arbitrary units the level of mental fatigue that was reported by players during a specific padel tournament (Díaz‐García, González‐Ponce, et al., 2021). Therefore, the negative impact of mental fatigue on padel‐specific performance that has been observed in the present study likely overestimates the negative impact that can be expected during a real competition (i.e., a lower degree of perceived level of mental fatigue has been observed in such a situation). However, currently, there is only one study that has analyzed the effects of a padel‐specific competition on mental fatigue (Díaz‐García, González‐Ponce, et al., 2021) and this study was performed only in qualifying rounds. Under normal conditions, matches of the main draw should be more mentally demanding than the matches played in qualifying rounds. As such, to properly evaluate whether the level of lab‐based induced mental fatigue and the level of mental fatigue that is perceived during padel tournaments really differ and the negative impact of mental fatigue on padel‐specific performance during real competitions is overestimated by the present study, more studies on this topic are necessary.

### 4.2 Padel‐specific accuracy and speed

The results of the present study showed that the performance of youth‐elite padel players significantly decreased in the mental fatigue condition compared to the control condition. Specifically, the accuracy of all investigated strokes (i.e., the drive, drive volley, bandeja and drive attack stroke after the use of the glass) was impaired. To the best of our knowledge, this is the first study analyzing the effects of mental fatigue on padel‐specific accuracy performance in youth‐elite male players. However, the present results are in line with similar studies that have previously been performed in table tennis (Le Mansec et al., 2018), cricket (Veness et al., 2017) and soccer (Smith, Coutts, et al., 2016; Smith, Zeuwts, et al., 2016), where the accuracy of sport‐specific movements was also impaired by acute mental fatigue. It is suggested that mental fatigue resulted in an adaptation of the athlete's sport‐specific motor control (Le Mansec et al., 2018). Specifically, the authors explained that the participants decreased the speed of the shots and movements to attempt to maintain the shot accuracy. This information is in line with the results of the present study for youth‐elite male padel players. Compared to in the control condition, these padel players decreased their shot speed in the mental fatigue condition, probably in an attempt to uphold their padel‐specific accuracy (i.e., the main performance measure in the present study) as much as possible. However, the decrease in shot speed was insufficient to uphold an optimal accuracy and behavioral carry‐over effects occurred from the 30‐min incongruent Stroop task to the padel‐specific performance task. Following the observation that mental fatigue also impairs padel‐specific performance, the importance of the quest for the mechanisms underlying the mental fatigue‐associated drop in sport‐specific psychomotor performance is emphasized again (Habay, Van Cutsem et al., 2021). One of the hypotheses is that the mental‐fatigue associated decrease in shot speed might be related to a change in motor control (Habay, Van Cutsem et al., 2021; Le Mansec et al., 2018). Based on findings of Rozand et al. (2015), Le Mansec et al. (2018) put forward that mental fatigue might alter the preparatory state of the movement, leading to an alteration of the motor command. Though, up to date, this hypothesis is still speculative, previous studies provide support for it. For example, Bray et al. (2008) indicated that sedentary undergraduates showed a significant degradation on maximal isometric endurance and higher electromyographic activation in a trial performed after a cognitive task when compared with a baseline trial. In addition, prolonged motor imagery has also been shown to change the mental state of the executors, the activity of motor areas and movement‐related cortical potentials (Jacquet, Lepers, et al., 2021; Jacquet, Poulin‐Charronnat, et al., 2021; Rozand et al., 2016). Habay, Proost, et al. (2021) confirmed that mental fatigue impairs the performance of table tennis players, while adding that brain activity (i.e., decreases in upper *α* band and *θ* band spectral power) also changed during the visuomotor task in the mentally fatigued condition. Van Cutsem et al. (2022) very recently conducted a detailed study into the occurrence of behavioral, perceived and neurophysiological carry‐over effects from one cognitive task to another. In the present study, we unfortunately did not include a measure of parasympathetic activity, glucose consumption or brain activity. Nevertheless, it is clear that we did not observe a time‐on‐task effect that is not related to the content/load of the first task in the present study (i.e., padel‐specific performance only dropped after the Stroop task and not after the documentary). In addition, the behavioral carry‐over effect that we found (i.e., performing a Stroop task resulted in a drop in padel‐specific performance) is associated with a greater degree of perceived mental fatigue. This association could, in light of the second mechanism that was determined in the study of Van Cutsem et al. (2022) (i.e., a decrease in response inhibition‐associated brain activity in corpus callosum, somatosensory association cortex and anterior circulate cortex; Van Cutsem et al. (2022)), suggest that the mental fatigue‐associated drop in padel‐specific performance was also associated with specific brain activity changes. However, because no brain activity measures were included in the present study, we cannot confirm this speculation. All the above‐mentioned results indicate that including neurophysiological measures in mental fatigue‐research can provide valuable additional insights and support the credibility of the hypothesis that an alteration of the motor command underlies the mental fatigue‐associated drop in sport‐specific psychomotor performance.

### 4.3 Limitations, future guidelines

The present study strengths include the ecological validity of the instrument used to quantify padel‐specific performance, and the large number of participants and measures involved. However, in future studies, authors should try to (i) use electroencephalography to elucidate the brain mechanisms that underlie the mental fatigue‐associated drop in sport‐specific psychomotor performance, (ii) include factors such as age, sex or physical fitness‐level that may provide additional insights in the interindividual variability in the impact of mental fatigue on padel‐specific performance, and (iii) include more padel‐specific measures of reaction time and cognitive processing and/or (iv) included more ecologically valid tasks to induce mental fatigue.

### 4.4 Practical applications

The presence of mental fatigue during official padel competitions has been previously confirmed (Díaz‐García, González‐Ponce, et al., 2021). Specifically, the authors reported that an increase in mental fatigue from pre‐ to post‐ padel matches was observed during a professional competition, with also an accumulation of mental fatigue between matches played on the same day. In line, the present study reports that increased levels of mental fatigue impair padel‐specific accuracy and speed in youth‐elite male players. This might of course impact the outcome of the match, as the number of forced and unforced errors or the speed of the shots are important indicators of padel‐specific performance (Courel‐Ibáñez et al., 2017). This information highlights the need for coaches and staff to consider the amount of mental fatigue generated during padel trainings and matches. Different practical applications for coaches could be suggested.

First of all, this information highlights the need to include measures of mental fatigue during training sessions and competitions. This could indicate the need to intervene as Russell et al. (2022) pointed out. Secondly, coaches should consider the mentally fatiguing nature of the trainings. In general, Van Cutsem and Marcora (2021) mention the following recommendations for practitioners in sport to cope with mental fatigue: (i) to avoid the presence of mentally fatiguing tasks close to competitions, (ii) to train the tolerance to mental fatigue so that performance is maintained in the mentally fatigued athlete and (iii) to use strategies to counter the effects of mental fatigue on performance. Even though it is an important issue, there is very little information available about how mentally fatiguing padel training sessions are (Rubio‐Morales et al., 2022). However, it has been reported that the presence of constraints (e.g., external rewards) that significantly increase the motivation of the padel players during training contributes to significant increases in the level of effort and mental fatigue (Díaz‐García, López‐Gajardo, et al., 2021). Therefore, it might be helpful for players that coaches include training sessions with a high cognitive load to develop a tolerance to mental fatigue. There is more information published on the mentally fatiguing nature of different constraints in soccer (Cárdenas et al., 2015; Díaz‐García, Pulido, et al., 2021; García‐Calvo et al., 2019, 2021). These outcomes could be partially translated to padel, and the staff employed in padel could then also use this to improve player performance. To prevent mental fatigue, coaches should simply limit the mental efforts of players during and after trainings that are performed close to competitions. Players should also avoid performing mentally fatiguing tasks close to competitions (e.g., the use of smartphones (Gantois et al., 2021)). Different countermeasures, such as caffeine, music and extrinsic motivation that coaches could use to counteract mental fatigue when it eventually occurs are also available (Proost et al., 2022). Another possible option is the use of the Brain Endurance Training, which is a training method where coaches increase the mental demands of their training (e.g., by the use of tasks such as the incongruent Stroop) with the purpose to develop specific adaptations against mental fatigue (Dallaway et al., 2021; Staiano et al., 2022).

## 5 CONCLUSIONS

This study demonstrates that acute mental fatigue impairs the accuracy of padel‐specific strokes such as drive, drive volley, bandeja and drive attack stroke after the use of the glass in youth‐elite male players. This level of mental fatigue also caused a decrease in the speed of the shots in this population, suggesting that an alteration of the motor command underlies the mental fatigue‐associated drop in padel‐specific psychomotor performance. Induced mental fatigue also impaired the Stroop task performance and the PVT reaction time of these players. The present study highlights the need to consider the detection, prevention and countering of mental fatigue in youth‐elite male padel players.

## CONFLICT OF INTEREST STATEMENT

Authors declare no conflict of interest.
